# Double-Hybrid DFT Functionals for the Condensed Phase: Gaussian and Plane Waves Implementation and Evaluation

**DOI:** 10.3390/molecules25215174

**Published:** 2020-11-06

**Authors:** Frederick Stein, Jürg Hutter, Vladimir V. Rybkin

**Affiliations:** Department of Chemistry, University of Zurich, Winterthurerstrasse 190, 8057 Zurich, Switzerland

**Keywords:** density functional theory, double-hybrid functionals, benchmark, wave-function correlation method

## Abstract

Intermolecular interactions play an important role for the understanding of catalysis, biochemistry and pharmacy. Double-hybrid density functionals (DHDFs) combine the proper treatment of short-range interactions of common density functionals with the correct description of long-range interactions of wave-function correlation methods. Up to now, there are only a few benchmark studies available examining the performance of DHDFs in condensed phase. We studied the performance of a small but diverse selection of DHDFs implemented within Gaussian and plane waves formalism on cohesive energies of four representative dispersion interaction dominated crystal structures. We found that the PWRB95 and ωB97X-2 functionals provide an excellent description of long-ranged interactions in solids. In addition, we identified numerical issues due to the extreme grid dependence of the underlying density functional for PWRB95. The basis set superposition error (BSSE) and convergence with respect to the super cell size are discussed for two different large basis sets.

## 1. Introduction

Electronic structure calculations for realistic condensed-phase systems are generally more involved than those for molecules. The former include more atoms and are performed under periodic boundary conditions (PBC), implying interactions between periodic images. Therefore, condensed-phase electronic structure modelling often relies on simple approximations. Tight-binding approaches—semiempirical methods, density functional based tight-binding (DFTB)—used to be the work horse in the field. With increased computational power Kohn-Sham density functional theory (KS DFT) [1] became a standard approach. Recently, implementations of wave function theories (WFT) became available, although their application is far from routine.

In DFT, energy is given as unique functional of electron density alone (Hohenberg-Kohn theorem) [2]. Although the exact functional is unknown, several approximation levels are available, often classified as rungs of the Jacob’s ladder of accuracy [3]. The most simple approximation includes only local information on the density (local density approximation, LDA) [4,5,6,7,8]. More eleborate theories take more properties of the density into account. Including the density gradient yields generalized-gradient (GGA) approximations (LYP [9], PBE functionals [10]), whereas including the kinetic energy density gives meta-GGA functionals [11,12,13] (e.g., TPSS functional [14]).

Incorporating a portion of exact exchange (non-local) leads to hybrid functionals (e.g., PBE0 [15], B3LYP [16]). Exact exchange energy is not density-dependent, but is rather a non-local quantity (dependent on the density matrix) borrowed from WFT, *viz.* from Hartree-Fock (HF) theory [17,18]. Hence, the term “hybrid” functional means including quantities from WFT, i.e., the Hartree-Fock exchange energy, into DFT functionals. Further examples of this approach are range-separated methods (HSE [19], WB97X [20]) and double-hybrid functionals, the latter can also involve range separation. Whereas hybrid functionals depend on the occupied KS orbitals, double-hybrid functionals include additionally virtual orbitals. They account for electron correlation in both DFT fashion via exchange-correlation functional and WFT fashion via excited determinants. We will refer to WFT methods which include correlation energy as Wave-function correlation (WFC) method.

Double-hybrid functionals [21,22,23,24,25,26] can potentially take “the best of the two worlds”. GGA-, meta-GGA- and hybrid DFT functionals are relatively fast and accurate for covalently and ionically bound systems. However, they intrinsically fail to describe long-range dispersion interactions (which is often coped with by explicit dispersion corrections [27,28,29,30,31,32] and non-local functionals [33,34]) and strong correlations. WFC methods, on the other hand, inherently include the correct asymptotic $R^{-6}$ behaviour. Their significant disadvantage is the high computational cost: $N^{4}$ scaling and higher in the canonical formulations. Reduced-cost methods allow decreasing the scaling, although with high prefactors. Consequently, the cost of a double-hybrid DFT calculation is defined by the cost of its WFT part. The question may then arise: why not use pure WFT instead of double-hybrid functionals? The answer is that the $N^{4}$–$N^{5}$ scaling of WFT methods used for double-hybrid functionals (second-order Møller-Plesset perturbation theory, MP2 [35]; random phase approximation, RPA [36]) are relatively crude approximations, and despite capturing long-range interactions they can be outperformed by DFT functionals. Thus, inclusion of electron correlation in WFT and DFT fashion may lead to the improved accuracy of both at moderate price as compared to highly precise WFT approaches, such as coupled-cluster methods [37] scaling as $N^{6}$ and higher.

Most condensed-phase implementations of electronic structure methods are based either on the use of plane waves (PW) or Gaussian basis sets. Plane waves constitute a basis in a strict mathematical sense: they are orthogonal and complete. In PW basis DFT and correlation energies converge systematically with basis size [38]. However, due to the fact that PW do not reflect the character of chemical bonding, a larger number of basis functions is needed for accurate calculations, which is detrimental for calculations with WFC methods as virtual space becomes huge. Since atom-centered Gaussian functions reasonably approximate atomic orbitals, good accuracy can be achieved with compact basis sets, i.e., at a lower computational price, especially for DFT. WFC energies are more sensitive to basis set size and exhibit slow convergence with basis set size [39], especially for long-range dispersion interactions [40]. WFC methods and DHDFs are available for PW basis sets in VASP [38,41], for Gaussian basis sets in CP2K [42], CRYSTAL [43] and GAMESS (US) [44] and for Slater type basis sets in ADF [45].

## 2. Theoretical Background

In the following, a,b,⋯ are virtual orbital indices, i,j,⋯ occupied orbital indices, p,q,⋯ general orbital indices, and P,Q,⋯ auxiliary function indices. In DFT, the total energy is given as a functional of the total ground-state density $n(\vec{r})$:

(1) $$E_{DFT}\left[n\right]=T_{0}\left[n\right]+E_{ne}\left[n\right]+E_{H}\left[n\right]+E_{XC}\left[n\right],$$

where $E_{DFT}\left[n\right]$ is the total energy functional, $T_{0}\left[n\right]$ is the kinetic energy of a reference system of non-interacting electrons, $E_{ne}\left[n\right]$ is the nuclei-electron interaction energy, $E_{H}\left[n\right]$ is the Hartree energy describing the classical electron-electron interaction energy, and $E_{XC}\left[n\right]$ is the exchange-correlation energy describing the quantum mechanical contributions of the electron-electron interaction. The ground-state density is expressed in terms of orbital functions $\psi_{i}\left(\vec{r}\right)$

(2) $$n\left(\vec{r}\right)=\sum_{i}{\left|\psi_{i}\left(\vec{r}\right)\right|}^{2}$$

where *i* runs over all occupied orbitals. The orbital functions fulfill the orthonormality constraint:

(3) $$\int d^{3}r\psi_{i}^{*}\left(\vec{r}\right)\psi_{j}\left(\vec{r}\right)=\delta_{ij}$$

with the Kronecker delta $\delta_{ij}$. The orbitals are solutions of the Kohn-Sham (KS) equation:

(4) $$(-\frac{\Delta }{2}+v_{ne}\left(\vec{r}\right)+v_{H}\left[n\right]\left(\vec{r}\right)+v_{XC}\left[n\right]\left(\vec{r}\right))\psi_{i}\left(\vec{r}\right)=\epsilon_{i}\psi_{i}\left(\vec{r}\right)$$

with the potential arising from the nuclei $v_{ne}\left(\vec{r}\right)$, the Hartree potential $v_{H}\left[n\right]\left(\vec{r}\right)$, the exchange- correlation (XC) potential $v_{XC}\left[n\right]\left(\vec{r}\right)$, and the orbital energy $\epsilon_{i}$ of orbital *i*. In this article, we will consider Gaussian functions centered at the atoms only.

Because the total energy functional is not known explicitly in terms of the ground-state density, we rely on approximations of the XC functional. These approximate energy functionals are given as integrals of a function explicitly depending on the ground-state density, its gradient and its Laplacian. For convenience, the XC functional is split into an exchange functional $E_{X}\left[n\right]$ and a correlation functional $E_{C}\left[n\right]$.

The more complex hybrid density functionals (HDFs) [16] include explicit information of the occupied orbitals. They modify the exchange functional by including a certain amount $\alpha_{X,HF}$ of Hartree-Fock (HF) exchange $E_{X,HF}\left[n\right]$ providing the exchange functional

(5) $$E_{X,hybrid}=\alpha_{X,HF}E_{X,HF}\left[n\right]+\alpha_{X,DFT}E_{X,DFT}\left[n\right].$$

We introduce the amount $\alpha_{X,DFT}$ of DFT exchange $E_{X,DFT}\left[n\right]$ to reflect that the DFT exchange functional is an already known GGA or meta-GGA functional (compare [15,16]). Using the Mulliken notation (chemists’ notation) for electron repulsion integrals

(6) $$\left(pq|rs\right)=\int d^{3}r\int d^{3}r^{\prime }\frac{\phi_{p}^{*}\left(\vec{r}\right)\phi_{q}\left(\vec{r}\right)\phi_{r}^{*}\left(\vec{r}{}^{\prime }\right)\phi_{s}\left(\vec{r}{}^{\prime }\right)}{\left|\vec{r}-\vec{r}{}^{\prime }\right|}$$

the HF exchange energy can be written as

(7) $$E_{X,HF}=-\frac{1}{2}\sum_{ij}\left(ij|ji\right).$$

Non-HDFs suffer from self-interaction errors [46]. These are reduced in HDFs but usually not fully cancelled since $\alpha_{X,HF}\neq 1$ in general case. This self-interaction error results in erroneous description of charge-separation processes and transition states. But even hybrid methods and HF lack a reasonable description of dispersion interactions decaying like $R^{-6}$ with *R* being a measure of charge separation.

For increased flexibility, we can further split the exchange functional in a long-range and a short-range functional and describe both with a given mixture of HF theory and DFT resulting in range-separated HDFs [19].

The highest flexibility is achieved by including virtual orbitals $\psi_{a}\left(\vec{r}\right)$. Double-hybrid density functionals (DHDFs) are HDFs in which the correlation functional is composed of a mixture of a DFT correlation functional $E_{C,DFT}\left[n\right]$ with ratio $\alpha_{C,DFT}$ and correlation energy $E_{C,WFT}\left[n\right]$ of a WFC method with ratio $\alpha_{C,WFT}$ providing a functional

(8) $$E_{C,double-hybrid}=\alpha_{C,WFT}E_{C,WFT}\left[n\right]+\alpha_{C,DFT}E_{C,DFT}\left[n\right].$$

Because WFC methods are computationally more demanding than HDFs or standard DFT functionals, most DHDFs exploit the MP2 theory, the SOS-MP2 theory, or the RPA method.

The correlation energy within the MP2 theory for closed-shell systems is

(9) $$E_{C,MP2}=\sum_{ijab}\frac{\left(ia|jb\right)[2(ia|jb)-(ib|ja)]}{\epsilon_{i}+\epsilon_{j}-\epsilon_{a}-\epsilon_{b}}.$$

The computationally most expensive step of the MP2 method is given by the transformation of the electron interaction integrals from atom orbital basis to molecular orbital basis leading to a $O(N^{5})$ scaling with *N* being a measure of system size. The prefactor can be reduced by the resolution-of-the-identity (RI) approach introducing an auxiliary basis in which densities are expanded giving the equation

(10) $$\left(pq|rs\right)=\sum_{P}B_{P}^{pq}B_{P}^{rs}$$

with

(11) $$\begin{array}{cc} B_{P}^{ia} & =\sum_{Q}\left(pq|Q\right){\left(Q|P\right)}^{-1/2} \end{array}$$

(12) $$\begin{array}{cc} \left(pq|P\right) & =\int d^{3}r\int d^{3}r^{\prime }\frac{\phi_{p}^{*}\left(\vec{r}\right)\phi_{q}\left(\vec{r}\right)\phi_{P}\left(\vec{r}{}^{\prime }\right)}{\left|\vec{r}-\vec{r}{}^{\prime }\right|} \end{array}$$

(13) $$\begin{array}{cc} \left(P|Q\right) & =\int d^{3}r\int d^{3}r^{\prime }\frac{\phi_{P}\left(\vec{r}\right)\phi_{Q}\left(\vec{r}{}^{\prime }\right)}{\left|\vec{r}-\vec{r}{}^{\prime }\right|}. \end{array}$$

This method is called RI-MP2 [47,48].

A simplified version of the RI-MP2 method is the Scaled-Opposite-Spin(SOS)-MP2 method [49] given by

(14) $$E_{C,SOS-MP2}=-\int_{0}^{\infty }d\tau \mathrm{Tr}(Q_{SOS-MP2}\left(\tau \right)Q_{SOS-MP2}^{T}\left(\tau \right))$$

with

(15) $${\left(Q_{SOS-MP2}\left(\tau \right)\right)}_{PQ}=\sum_{ia}B_{P}^{ia}e^{\tau (\epsilon_{i}-\epsilon_{a})}B_{Q}^{ia}.$$

The integration is carried out numerically using a Minimax quadrature. The RI-SOS-MP2 method scales like $O(N^{4})$.

Another correlation method with increasing popularity is the Random Phase Approximation (RPA) method [50,51] within the RI approximation

(16) $$E_{C,RPA}=\frac{1}{2}\int_{0}^{\infty }\frac{d\omega }{2\pi }\mathrm{Tr}(\ln\left(1+Q_{RPA}\left(\omega \right)\right)-Q_{RPA}\left(\omega \right))$$

with

(17) $$Q_{RPA}\left(\omega \right)=2\sum_{ia}B_{P}^{ia}\frac{\epsilon_{a}-\epsilon_{i}}{\omega^{2}+{\left(\epsilon_{a}-\epsilon_{i}\right)}^{2}}B_{Q}^{ia}.$$

RI-RPA scales like $O(N^{4})$. As with the RI-SOS-MP2 method, the integration is carried out numerically using a Clenshaw-Curtis grid [52] or a Minimax grid [53,54].

All WFC methods and all DHDFs correctly reproduce the $R^{-6}$ energy behaviour of long-range interactions. Comparable to range-separated HDFs, there are DHDFs with range-separated exchange functionals like the ωB97X-2 functional [55]. Further, there are DHDFs with range-separated correlation functionals [56]. In this article, we will not focus on DHDFs with range-separated correlation functionals and refer to the literature [40,57,58,59,60] for more details.

## 3. Computational Details

### 3.1. Gaussian and Plane Waves Method (GPW) and Integral Evaluation

The Gaussian and plane waves method (GPW) [61] allows for efficient periodic calculations with Gaussian basis sets using a dual representation of the electronic density and molecular orbitals. It assumes the use of a primary Gaussian basis for the expansion of matrix quantities (density matrix, KS matrix) and an auxiliary plane waves (PW) basis for the evaluation of the Hartree potential and the numerical integration of density functionals. To converge GPW calculations, one has to pay attention to both the size and quality of the Gaussian basis and the energy cutoff for the PWs. In the current implementation, GPW is used for the calculation of the Hartree potential, XC functionals, and two and three center integrals necessary for the RI-MP2 and RI-RPA methods. Exchange integrals are computed analytically using a truncated Coulomb potential [62].

### 3.2. Test Systems

Because we are interested in the description of intermolecular interactions, we are testing the functionals on molecular crystals (NH3, HCN) and rare-gase crystals (Ar, Ne) which have been studied by Sansone et al. [63]. Structural information of the unit cells are summarized in Table 1. In case of molecular solids, we were using structures reoptimized at the B3LYP-D* level [64]. Molecular crystals represent systems with a mixture of covalent bonding and dispersion interactions. NH_3_ and HCN crystals additionally contain hydrogen bonds which are crucial for the discription of proteins. In contrast to that, there are only dispersion interactions within the rare-gas crystals. This results in low cohesive energies and the need for well-balanced functionals.

### 3.3. Parameters of the Calculations

All calculations have been carried using a development version 8.0 of CP2K [42]. To ensure convergence with respect to the density cutoff, we were using high cutoffs of 1500 *Ry* for all RPA and MP2 calculations, 4000 *Ry* for remaining calculations of the molecular crystals NH_3_ and HCN, and 10,000 *Ry* for the rare-gas crystals Ar and Ne (see Section 4.1 for more details) and a relative cutoff of 50 *Ry*. For the rare-gas crystals, we set the parameters EPS_DEFAULT, EPS_PGF_ORB, EPS_SCF, and EPS_SCHWARZ in the HF section to 10^−30^, 10^−50^, 10^−5^, and 10^−10^, respectively, for the molecular crystals, we were using for the same parameters 10^−20^, 10^−40^, 10^−5^, and 10^−9^, respectively (see the CP2K manual for the meaning of these parameters). HF calculations for the bulk systems were using a truncated Coulomb potential with a cutoff radius of roughly half the super cell size. All densities have been smoothed using the NN10 method.

RI-MP2, RI-SOS-MP2 and RI-RPA calculations have been carried out using the GPW method to determine all integrals with a primary cutoff of 300 *Ry* and a relative cutoff of 50 *Ry*. We have exploited an 8-point minimax grid for all RI-RPA and RI-SOS-MP2 calculations.

### 3.4. Choice of Functionals and Implementation

We carried out calculations at the PBE [10], ωB97M-V [72], ωB97X-2 [55], PW6B95 [73], PWRB95 [74], SOS-PBE0-2 [75], RI-MP2 [35,47] and RI-RPA [51] levels of theory. PBE and RI-MP2 are used to compare differences between valence-only calculations of our valence-only calculations and the all-electron calculations of Sansone et al. [63]. PW6B95 is a meta-hybrid functional which performed best for weakly interacting systems with more pronounced dispersion interactions. PWRB95 is its RPA-based DHDF. ωB97M-V is a dispersion-corrected range-separated meta-hybrid functional. ωB97X-2 is an MP2-based DHDF with range-separated exchange functional. SOS-PBE0-2 is a RI-SOS-MP2-based DHDF. With this choice, we cover a large variety of different flavours of meta-hybrid and DHDF theories. Due to very high computational cost, we have restricted ourselves to this small, but representative set of functionals: one for each flavour of DHDFs and a corresponding HDF.

PBE calculations have been carried out using the CP2K implementation of PBE. For the ωB97M-V, and the PW6B95 functionals, we exploited the implementations of the LibXC library [76], version 4.3.4. Since the VV10 dispersion correction is not available in CP2K, we relied on the rVV10 correction and the parametrization suggested by Mardirossian et al. [77]. For the ωB97X-2 and PWRB95 functionals, we implemented the required parameter sets into the LibXC library.

### 3.5. Basis Sets and Pseudopotentials

The MP2 and RPA implementations within CP2K rely on a pseudopotential (PP) approach with Goedecker-Teter-Hutter PPs [78]. For the PBE functional, we used PPs optimized for PBE, for RPA and MP2 calculations, we were using PPs optimized for HF whereas for both HDFs and all DHDFs, we utilized PPs optimized for the PBE0 functional. All PPs have been taken from the Github repository of Jürg Hutter [79].

Correlation-consistent primary basis sets and suitable auxiliary basis sets of double zeta (DZ) and triple zeta (TZ) quality for the elements C, H, N and O have been taken from Del Ben et al. [80] We have optimized appropriate correlation-consistent primary and auxiliary basis sets of the same qualities for Ne and Ar using the polarization functions of the respective Dunning basis sets [48,81,82]. All PPs and primary and auxiliary basis sets are compiled in the Appendix A, Appendix B and Appendix C.

### 3.6. Cohesive Energies and Basis Set Superposition Error

To determine total energies per formula unit, we carried out calculations of 2×2×2, 3×3×3 and 4×4×4 supercells of all given unit cells and used a linear fit of the total energy per formula unit against the inverse of the cell volume.

Because calculations of cohesive energies usually suffer from basis set superposition errors (BSSE), we perform a counterpoise correction [83]. The BSSE-free cohesive energies $E_{coh}$ are calculated according to

(18) $$\begin{array}{cc} E_{coh} & =E_{bulk}-E_{mol+ghost,bulk}+E_{mol,bulk}-E_{mol,gas} \end{array}$$

with the total bulk energy per formula unit extrapolated to infinite cell volume $E_{bulk}$, the energy of the molecule with ghost atoms $E_{mol+ghost,bulk}$, the energy of the molecule using the bulk geometry $E_{mol,bulk}$, and the total energy of the molecule using an optimized gas phase structure $E_{mol,gas}$. For Ar and Ne, we trivially have $E_{mol,bulk}=E_{mol,gas}$.

The corresponding BSSE is given by

(19) $$\begin{array}{c} \Delta E_{BSSE}=E_{mol+ghost,bulk}-E_{mol,bulk}. \end{array}$$

For the BSSE calculations, we took the crystalline structures, chose one molecule (or atom for Ar and Ne) surrounded by all ghost atoms within a 3×3×3 supercell.

## 4. Results

### 4.1. General Remarks

We found the convergence of total energies of meta-HDFs PW6B95 and PWRB95 requires very tight energy cutoffs for the auxiliary PW basis of at least 4000 *Ry*. In contrast to that, calculations with the other meta-HDFs in our benchmark study, ωB97M-V, provided reasonable results with a cutoff of only 1200 *Ry*. Because the basis functions for the elements argon and neon are more localized than those for hydrogen, carbon and nitrogen, higher cutoffs for the noble gases were needed for an adequate representation of the basis functions of these elements on the grid.

It is well-known that GGA functionals and especially meta-GGA functionals require very tight integration grids for convergence and thus accurate results. Such cutoffs reflect numerical issues and the need for very fine integration grids when using the PW6B95 and PWRB95 functionals. Such grids are not necessary for the ωB97M-V functional which has been optimized with coarser integration grids in mind [72]. Thus, energy differences converged faster with ωB97M-V and PBE. Nevertheless, the total energies were not converged. To remove any possible problems due to incomplete convergence with respect to cutoffs, we utilized unusually high cutoffs for all density functionals.

Furthermore, we have found convergence problems with the PW6B95 and PWRB95 functionals, which can be resolved with density smoothing. Unfortunately, in some cases an increase of the energy cutoff for the density resulted in SCF convergence issues which could not be resolved with tighter filter thresholds. Nevertheless, we were able to achieve convergence by restarting the calculations with a higher cutoff starting from the converged SCF results with a lower cutoff. This was not possible for argon, where we exploited a cutoff of 4000 *Ry* for the PW6B95 and PWRB95 functional. Thus, some numbers for the PW6B95 and PWRB95 functionals are not fully converged with respect to the density cutoff.

Due to the higher computational costs, we have not carried out calculations of the 4×4×4 supercells on the TZ level.

All cohesive energies are compiled in Table 2 and Table 3.

### 4.2. Convergence with Respect to Super Cell Size

**Figure 1:**
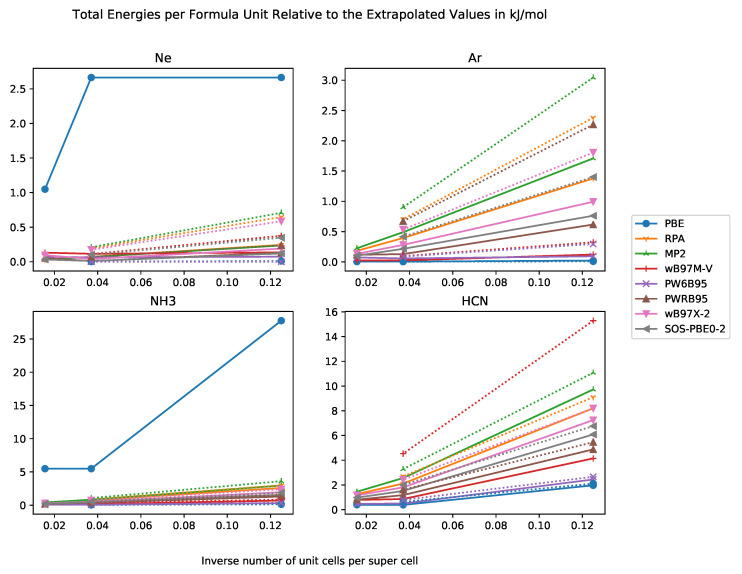
Total energies per formula unit relative to extrapolated total energy in kJ·mol^−1^ against inverse number of unit cells in supercell with basis sets of DZ and TZ quality for the systems Ne, Ar, NH_3_, HCN.

In Figure 1, we compiled the differences in total energies per formula unit relative to the extrapolated total energies. In general, we expect the total energies to decrease with increasing supercell size and the extrapolated value is a lower bound for the total energies of the super cells. Our results show exactly this behaviour.

An important question is for which supercell size the error becomes negligible. A useful magnitude is given by the chemical accuracy of 4 kJ·mol^−1^. For weakly-interacting systems such as rare-gas crystals with cohesive energy of less than chemical accuracy, the order of magnitude is set by the cohesive energy itself. As the error of a method should be not larger than chemical accuracy, the allowed error of the supercell method must be at least one order of magnitude smaller then the methodological error, i.e., not larger than 0.4 kJ·mol^−1^. We find that a 3×3×3 super cell provides sufficient accuracy for all functionals and test systems. This behaviour is in agreement with the literature [80]. Sometimes, the total energy per formula unit of the 4×4×4 super cell has a higher magnitude than this of the 3×3×3 supercell, which may be due to numerical issues. For PBE, a cubic fit does not seem to be appropriate, and an exponential fit should be used instead.

### 4.3. Convergence of the BSSE

**Figure 2:**
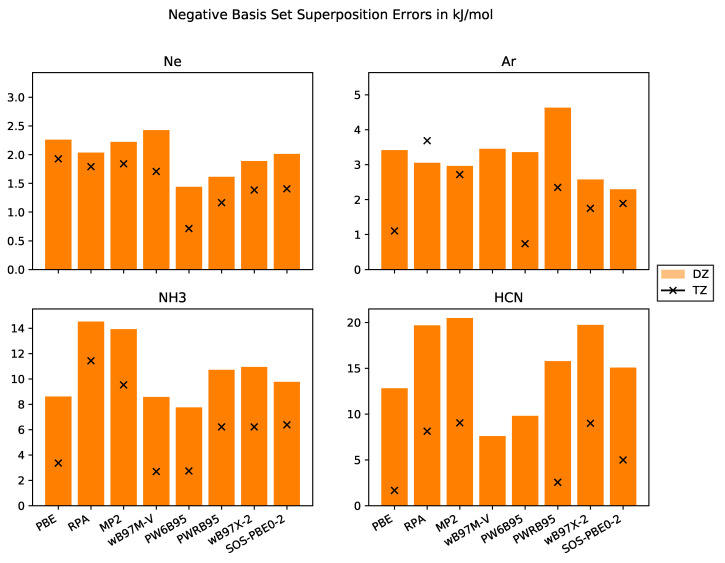
Negative Basis set superposition errors in kJ·mol^−1^ with basis sets of DZ and TZ quality for the systems Ne, Ar, NH_3_, HCN.

The BSSEs for the different test systems are compiled in Figure 2. First, we would like to point out that the BSSE is significantly larger for the molecular crystals than for the rare-gas crystals. This might be related to the larger number of atoms per molecule and to the spread of the basis functions. Since the effective core charge of rare-gas atoms is larger than for carbon or nitrogen, the basis functions are more localized which results in weaker overlap with neighbouring atoms. This is supported by the smaller reduction in BSSE for Ar and Ne when we exploit larger basis sets. Thus, augmentation of basis sets must significantly reduce BSSEs of Ar and Ne. Indeed, diffuse basis functions actually improve cohesive energies as shown by Sansone et al. [63].

Molecular crystals are thus more suitable objects to study BSSE than rare-gas crystals. For both molecular crystals in the test set, the non-DHDFs PBE, PW6B95 and ωB97M-V, provide the smallest BSSEs whereas the two WFC methods MP2 and RPA have the largest BSSEs, as expected. The DHDFs have a BSSE between both classes of methods because they employ a mixture of DFT and WFC methods.

Furthermore, we note that the WFC methods in CP2K are implemented within the RI approximation employing an auxiliary basis set. This leads to an additional source of BSSE for RPA, MP2 and all the DHDFs because the addition of the auxiliary functions of the ghost atoms increases the overall accuracy.

### 4.4. Convergence with Respect to Basis Set Size

**Figure 3:**
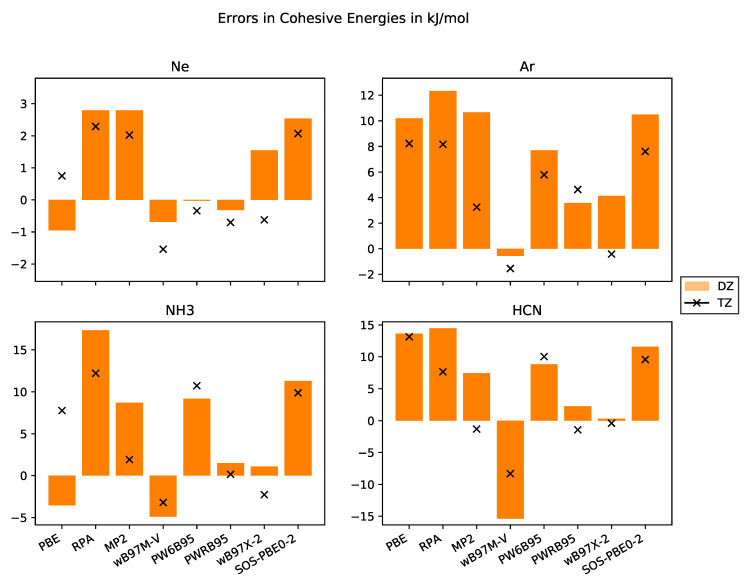
Errors in cohesive energies in kJ·mol^−1^ with respect to the experimental values with basis sets of DZ and TZ quality for the systems Ne, Ar, NH_3_, HCN.

In numerous studies, it was shown that total energies from DFT calculations converge exponentially with respect to basis set size. In contrast to that, total energies from WFC methods converge cubically with respect to basis set size when employing correlation-consistent basis sets. Thus, most DHDFs are expected to have a cubic convergence with respect to basis set size but with a smaller prefactor. DHDFs employing a long-ranged Coulomb operator only and describing short-ranged interactions with a density functional, converge exponentially [40]. This behaviour is confirmed with our data compiled in Figure 3.

Since larger basis sets systematically reduce total energies, cohesive energies increase. We observe this behaviour for the WFC methods and almost all DHDFs. The slight difference for PWRB95 in case of Ar may be due to not full convergence with respect to super cell size. For the other functionals—PBE, ωB97M-V and PW6B95—the cohesive energy from the TZ basis set is sometimes higher, i.e., the system is weaker bound. One problem might be that the 2×2×2 super cells are not yet fully converged or the extrapolation scheme using a linear fit of the total energies versus the inverse of the volume is not appropriate and an exponential fit might be more suitable.

Next, we would like to discuss the results obtained for the molecular crystals NH_3_ and HCN. They are bound together by covalent bonds, dipole-dipole interactions, and dispersion interactions. For both systems, the results with the RPA and MP2 methods significantly improve the results over GGA DFT functionals, MP2 even achieving chemical accuracy. The ωB97M-V functional also provides very accurate numbers. The PW6B95 functional, as PBE, systematically underestimates the cohesive energies with errors compatible to PBE. The PWRB95 functional significantly improves upon the results of its relative PW6B95, bringing them within 1 kJ·mol^−1^ from the experiment. The same holds for the ωB97X-2 functional compared with the ωB97M-V functional, although the DHDF is based on the non-meta-GGA HDF ωB97X [86]. One of the worst performing functionals is SOS-PBE0-2.

For the rare-gas crystals, the picture is more complicated because the absolute values of the cohesive energies are of the order of the chemical accuracy. As pointed out by Sansone et al. [63], augmented basis sets are required for these systems. Our cohesive energies from MP2 with a TZ basis set are only slightly lower than their result with a DZ basis set but still much worse than those with an augmented basis set for both Ne and Ar. Consequently, our results for Ne do not allow for an evaluation of the performance of these functionals and further studies employing either quadruple or augmented basis sets (which are to be constructed) are needed. Nevertheless, our results for Ar show that the ωB97X-2 functional provides a good description. The same holds for MP2, PW6B95 and PWRB95, although one needs further investigations with augmented basis sets.

This issue does not apply to the molecular crystals. Indeed, our cohesive energies with a TZ basis set are even lower than those with an augmented basis set. Thus, the use of augmented basis sets is not necessary for the molecular crystals. This result is important for reducing computational costs of HF calculations and low-scaling WFC methods.

## 5. Discussion

Because DHDFs can be considered to be a mixture of DFT and WFC methods, the flexibility of DHDF parametrizations can yield approaches more accurate than the parent DFT and WFC functionals. At the same time, they inherit the shortcomings of both classes. Due to the dependence on the grid parameters, the functionals PW6B95 and PWRB95 are more difficult to use than others: care must be taken to check whether the results are converged with respect to the grid parameters, in CP2K, the density cutoff.

As expected, PBE can only provide the order of magnitude for weakly interacting systems, although it converges fast with respect to basis set size and has a low BSSE. MP2 and RPA are more sensitive to the basis set size and exhibit large BSSEs. These methods provide a moderate accuracy for different systems with small basis sets.

Non-DHDFs benefit from lower BSSEs. The PW6B95 functional has high demands on integration grids. Both considered functionals also provide a moderate accuracy and should be favourable over MP2 and RPA with their higher computational costs.

The double-hybrid functionals PWRB95 and ωB97X-2 show excellent performance with moderate BSSEs and lower basis set incompleteness errors. Both have computational costs compatible to full MP2 or RPA calculations and inherit the need of fine integration grids for accurate results, especially for PWRB95.

The non-empirical SOS-MP2 based DHDF, SOS-PBE0-2, does not provide any advantage as compared to the original methods. It was pointed out by different authors [87,88] that non-empirical DHDFs usually perform worse than empirical DHDFs.

## 6. Conclusions

In this study, we examined a selection of different HDFs and DHDFs by computing cohesive energies in four different crystal structures. Our results show that DHDFs inherit the shortcomings of the underlying DFT functional (integration grids) and the underlying WFC method (computational costs, BSSE, basis set dependence). We were able to show that the PWRB95 and the ωB97X-2 functionals provide excellent accuracy for molecular and rare-gas crystals. The HDFs ωB97M-V and PW6B95 also provide reasonable accuracy for these systems, whereas the SOS-PBE0-2 functional underperforms and can not be recommended.

The exploited basis sets allow a good description of molecular crystals. For the rare-gas crystals, we showed that non-augmented basis sets are not sufficient to achieve energy convergence with respect to the basis set size. Due to the high computational costs, we leave studies with augmented basis sets (and the construction of those basis sets) as well as benchmarking more range-separated DHDFs for future prospect.

## Figures and Tables

**Table 1:** Structural information about the bulk structures used in this study. $n_{fu}$ is the number of formula units per unit cell. References for the geometrical information of the respective system are provided in the last column. Please note that there was a mistake in the cell parameters of CO_2_ provided in reference [63].

System	*a*;*b*;*c* (Å)	$n_{\mathrm{fu}}$	References
NH_3_	5.048	4	[65]
HCN	4.13; 4.85; 4.34	2	[66]
Ne	4.464	4	[67,68,69]
Ar	5.300	4	[70,71]

**Table 2:** Cohesion energies $E_{coh}$ and absolute relative error with respect to experimental results for all considered methods and systems in kJ/mol exploiting basis sets of DZ quality. The statistical indicators are the mean absolute error (MAE) and the mean absolute relative error (MARE). ^*a*^ This work. ^*b*^ Values by Sansonse et al. [63]. ^*c*^ Experimental values [68,71,84], corrected for zero-point energy (ZPE) and thermal effects at 298 K [63,85].

Functional	$E_{\mathrm{coh}}\left(\mathrm{Ne}\right)$	%	$E_{\mathrm{coh}}\left(\mathrm{Ar}\right)$	%	$E_{\mathrm{coh}}\left({\mathrm{NH}}_{3}\right)$	%	$E_{\mathrm{coh}}\left(\mathrm{HCN}\right)$	%	MAE	MARE
PBE ^*a*^	−2.92	48	2.47	131	−39.9	9	−29.0	32	7.09	0.55
PBE ^*b*^	−0.27	86	2.36	130	−26.9	25	−28.0	34	8.95	0.69
RPA ^*a*^	0.82	141	4.61	159	−19.0	47	−28.1	33	11.7	0.96
MP2 ^*a*^	0.83	141	2.95	138	−27.6	23	−35.2	17	7.40	0.80
MP2 ^*b*^	0.22	111	3.13	140	−24.2	33	−31.7	25	9.01	0.78
ωB97M-V ^*a*^	−2.65	34	−8.30	7	−41.2	13	−58.0	36	5.37	0.23
PW6B95 ^*a*^	−1.99	1	−0.03	99	−27.1	25	−33.8	20	6.43	0.37
PWRB95 ^*a*^	−2.29	16	−4.15	46	−34.8	4	−40.3	5	1.91	0.18
ωB97X-2 ^*a*^	−0.43	78	−3.60	53	−35.2	3	−42.3	0	1.77	0.34
SOS-PBE0-2 ^*a*^	0.57	128	2.76	135	−25.0	31	−31.0	27	8.98	0.81
Expt. ^*c*^	−1.97		−7.73		−36.3		−42.6			

**Table 3:** Same as Table 2, but with basis sets of TZ quality. ^*b*^ exploits basis sets of augmented DZ quality.

Functional	$E_{\mathrm{coh}}\left(\mathrm{Ne}\right)$	%	$E_{\mathrm{coh}}\left(\mathrm{Ar}\right)$	%	$E_{\mathrm{coh}}\left({\mathrm{NH}}_{3}\right)$	%	$E_{\mathrm{coh}}\left(\mathrm{HCN}\right)$	%	MAE	MARE
PBE ^*a*^	−1.22	37	0.49	106	−28.5	21	−29.5	30	7.47	0.49
PBE ^*b*^	−0.40	79	0.42	105	−26.2	27	−29.7	30	8.18	0.61
RPA ^*a*^	0.32	116	0.44	105	−24.1	33	−34.9	17	7.58	0.68
MP2 ^*a*^	0.05	102	−4.48	42	−34.4	5	−43.9	3	2.13	0.38
MP2 ^*b*^	−1.10	44	−6.45	16	−31.8	12	−41.4	2	1.96	0.19
ωB97M-V ^*a*^	−3.51	78	−9.28	20	−39.5	8	−50.9	19	3.65	0.32
PW6B95 ^*a*^	−2.31	17	−1.95	74	−25.6	29	−32.6	23	6.72	0.36
PWRB95 ^*a*^	−2.67	35	−3.10	59	−36.1	0	−44.0	3	1.73	0.25
ωB97X-2 ^*a*^	−2.59	31	−8.15	5	−38.6	6	−43.0	0	0.93	0.11
SOS-PBE0-2 ^*a*^	0.10	105	−0.12	98	−26.4	27	−33.0	22	7.28	0.63
Expt. ^*c*^	−1.97		−7.73		−36.3		−42.6			

## Abbreviations

The following abbreviations are used in this manuscript:

DFT	Density Functional Theory
DHDF	Double-Hybrid Density Functional
DZ	Double Zeta
GTO	Gaussian Type Orbital
HDF	Hybrid Density Functional
HF	Hartree-Fock
KS	Kohn-Sham
MP2	second order Moller-Plesset Perturbation theory
PW	Plane Wave
RI	Resolution of the Identity
RPA	Random-Phase Approximation
SOS-MP2	Scaled Opposite-Spin MP2
TZ	Triple Zeta
WFC	Wave-Function Correlation
WFT	Wave-Function Theory
XC	Exchange-Correlation

## Appendix A. Pseudopotentials

### Appendix A.1. PBE Pseudopotential

**Table A1:** PBE pseudopotential parameters. The format corresponds to [78]. Pseudopotentials are taken from [79].

H	1	1	
0.20059317301776	−4.17806832477260	0.72440924243368	
C	6	4	
0.33855479630051	−8.80455195420776	1.33837678314185	
0.30260967537284	9.62286249628669		
N	7	5	
0.28382600053810	−12.41517350030142	1.86813618209744	
0.25541754972811	13.63124869974610		
O	8	6	
0.24446328480160	−16.67548222363837	2.48908598241780	
0.22097110943471	18.33446866406285		
Ne	10	8	
0.19013599957922	−27.11394809602904	4.36380616652624	
0.17606810618356	28.17757050106910	0.83365740579601	−1.07616503335814
0.19546571702831	−0.23610573844687		
Ar	18	8	
0.40003082668805	−7.08796199095755		
0.31882882990174	17.25258480082010	−5.58549698978039	7.21031819907047
0.35335630753415	4.97482100660240		

### Appendix A.2. PBE0 Pseudopotential

**Table A2:** PBE0 pseudopotential parameters. The format corresponds to [78]. Pseudopotentials are taken from [79].

H	1	1	
0.20049539759096	−4.17780338804233	0.72403926676805	
C	6	4	
0.34015230644208	−8.75626046428525	1.33212403341974	
0.30255799930084	9.58980355283555		
N	7	5	
0.28405138134082	−12.39652421586226	1.86372383417056	
0.25538070446385	13.63073438324169		
O	8	6	
0.24671011902360	−16.65533253748591	2.50854752353111	
0.22100154713718	18.34370453997725		
Ne	10	8	
0.19050423878827	−27.40404160755363	4.42644219542327	0.00312528175949
0.17609378094694	28.18364816845336	0.83365182689679	−1.06378213860369
0.19427680906964	−0.23683812086750		
Ar	18	8	
0.39979462541098	−7.23417721420866	0.00616780402846	
0.31880599007091	17.21513221796928	−5.58548607072547	7.21072495408957
0.35343634803616	4.97384170460103		

### Appendix A.3. HF Pseudopotential

**Table A3:** Hartree-Fock pseudopotential parameters. The format corresponds to [78]. Pseudopotentials are taken from [79].

H	1	1	
0.20049539759096	−4.17780338804233	0.72403926676805	
C	6	4	
0.34816792458406	−8.54312820557867	1.33276540541946	
0.30230247000627	9.59710582360109		
N	7	5	
0.28300476743411	−12.39840200798251	1.86939057420079	
0.25539202567537	13.64483766978610		
O	8	6	
0.24676969870316	−16.66528269564613	2.52030687064467	
0.22121058101998	18.39425181647437		
Ne	10	8	
0.19050265092574	−27.39590696172339	4.41958869715540	0.01834396326683
0.17637388496062	28.18533818441574	0.83365996989179	−1.04842942962620
0.19585379054851	−0.27609661906079		
Ar	18	8	
0.39771927261258	−7.21348927487361	0.01323122557817	
0.31872450490949	17.20921819285275	−5.58549109340678	7.19978913165534
0.35357441343299	4.98951929408379		

## Appendix B. Primary Basis Sets

**Table A4:** Parameters of the cc-DZVP basis set of Hydrogen. Parameters taken from [80].

Shell Type	Exponents	Contraction Coefficients
s	8.3744350009	−0.0283380461
	1.8058681460	−0.1333810052
	0.4852528328	−0.3995676063
s	0.1658236932	1.0000000000
p	0.7270000000	1.0000000000

**Table A5:** Parameters of the cc-TZVP basis set of Hydrogen. Parameters taken from [80].

Shell Type	Exponents	Contraction Coefficients
s	10.8827241585	−0.0167058885
	3.0968750876	−0.0627538300
	0.9874518162	−0.1917521975
s	0.3450687533	1.0000000000
s	0.1492693554	1.0000000000
p	1.4070000000	1.0000000000
p	0.3880000000	1.0000000000
d	1.0570000000	1.0000000000

**Table A6:** Parameters of the cc-DZVP basis set of Carbon. Parameters taken from [80].

Shell Type	Exponents	Contraction Coefficients	
sp	4.3362376436	0.1490797872	−0.0878123619
	1.2881838513	−0.0292640031	−0.2775560300
	0.4037767149	−0.6882040510	−0.4712295093
sp	0.1187877657	1.0000000000	1.0000000000
d	0.5500000000	1.0000000000	

**Table A7:** Parameters of the cc-TZVP basis set of Carbon. Parameters taken from [80].

Shell Type	Exponents	Contraction Coefficients	
sp	5.3685662937	0.0974901974	−0.0510969367
	1.9830691554	0.1041996677	−0.1693035193
	0.6978346167	−0.3645093878	−0.3579933930
sp	0.2430968816	1.0000000000	1.0000000000
sp	0.0812865018	1.0000000000	1.0000000000
d	1.0970000000	1.0000000000	
d	0.3180000000	1.0000000000	
f	0.7610000000	1.0000000000	

**Table A8:** Parameters of the cc-DZVP basis set of Nitrogen. Parameters taken from [80].

Shell Type	Exponents	Contraction Coefficients	
sp	6.1526903413	0.1506300537	−0.0950603476
	1.8236332280	−0.0360100734	−0.2918864295
	0.5676628870	−0.6942023212	−0.4739050050
sp	0.1628222852	1.0000000000	1.0000000000
d	0.8170000000	1.0000000000	

**Table A9:** Parameters of the cc-TZVP basis set of Nitrogen. Parameters taken from [80].

Shell Type	Exponents	Contraction Coefficients	
sp	7.6227447102	0.0983924689	−0.0561654555
	2.7970605447	0.1045217098	−0.1798165209
	0.9909765447	−0.3742661352	−0.3653986185
sp	0.3417314862	1.0000000000	1.0000000000
sp	0.1116822743	1.0000000000	1.0000000000
d	1.6540000000	1.0000000000	
d	0.4690000000	1.0000000000	
f	1.0930000000	1.0000000000	

**Table A10:** Parameters of the cc-DZVP basis set of Oxygen. Parameters taken from [80].

Shell Type	Exponents	Contraction Coefficients	
sp	8.3043855492	0.1510165999	−0.0995679273
	2.4579484191	−0.0393195364	−0.3011422449
	0.7597373434	−0.6971724029	−0.4750857083
sp	0.2136388632	1.0000000000	1.0000000000
d	1.1850000000	1.0000000000	

**Table A11:** Parameters of the cc-TZVP basis set of Oxygen. Parameters taken from [80].

Shell Type	Exponents	Contraction Coefficients	
sp	10.2674419938	0.0989598460	−0.0595856940
	3.7480495696	0.1041178339	−0.1875649045
	1.3308337704	−0.3808255700	−0.3700707718
sp	0.4556802254	1.0000000000	1.0000000000
sp	0.1462920596	1.0000000000	1.0000000000
d	2.3140000000	1.0000000000	
d	0.6450000000	1.0000000000	
f	1.4280000000	1.0000000000	

**Table A12:** Parameters of the cc-DZVP basis set of Neon.

Shell Type	Exponents	Contraction Coefficients	
sp	13.8523672900	0.1501498200	0.10214300
	4.0685498000	−0.0314908700	0.3058092400
	1.2730584300	−0.7070497300	0.4766050400
sp	0.3565013600	1.0000000000	1.0000000000
d	2.2020000000	1.0000000000	

**Table A13:** Parameters of the cc-TZVP basis set of Neon.

Shell Type	Exponents	Contraction Coefficients	
sp	17.4276488400	0.073686700	0.0702714400
	6.3439264100	0.0969132500	0.2167849700
	2.2823205800	−0.3010470300	0.4317763300
sp	0.7945993700	1.0000000000	1.0000000000
sp	0.2560537300	1.0000000000	1.0000000000
d	4.0140000000	1.0000000000	
d	1.0960000000	1.0000000000	
f	2.5440000000	1.0000000000	

**Table A14:** Parameters of the cc-DZVP basis set of Argon.

Shell Type	Exponents	Contraction Coefficients	
sp	2.6724631600	0.1547491900	0.2663267700
	1.5750569800	−0.1300613000	−1.0821938600
	0.5528926600	−0.1247859600	0.1177549000
sp	0.1720724500	1.0000000000	1.0000000000
d	0.7380000000	1.0000000000	

**Table A15:** Parameters of the cc-TZVP basis set of Argon.

Shell Type	Exponents	Contraction Coefficients	
sp	3.5650652500	−0.03560400	−0.0341601300
	2.8711385000	0.107453000	0.0274003200
	0.928908200	−0.070935800	0.1084604500
sp	0.3762992800	1.0000000000	1.0000000000
sp	0.1388133000	1.0000000000	1.0000000000
d	1.2540000000	1.0000000000	
d	0.4100000000	1.0000000000	
f	0.8900000000	1.0000000000	

## Appendix C. Auxiliary Basis Sets

**Table A16:** Parameters of the cc-DZVP auxiliary basis set of Hydrogen. Parameters taken from [89].

Shell Type	Exponents	Contraction Coefficients
s	5.1153315245	1.0000000000
s	1.1472440266	1.0000000000
s	0.3203181150	1.0000000000
p	1.9149400132	1.0000000000
p	0.9859513111	1.0000000000
d	1.1714848284	1.0000000000

**Table A17:** Parameters of the cc-TZVP auxiliary basis set of Hydrogen. Parameters taken from [89].

Shell Type	Exponents	Contraction Coefficients
s	8.5115919487	1.0000000000
s	1.8744684087	1.0000000000
s	0.5632515602	1.0000000000
s	0.3698299759	1.0000000000
p	2.3711712242	1.0000000000
p	1.1794161391	1.0000000000
p	0.6050431621	1.0000000000
d	1.8092525711	1.0000000000
d	1.1433220615	1.0000000000
f	1.8065804513	1.0000000000

**Table A18:** Parameters of the cc-DZVP auxiliary basis set of Carbon. Parameters taken from [89].

Shell Type	Exponents	Contraction Coefficients
s	13.8045000000	1.0000000000
s	4.7727700000	1.0000000000
s	1.5133300005	1.0000000000
s	0.7826969986	1.0000000000
s	0.4090720022	1.0000000000
s	0.2067960415	1.0000000000
p	6.0052300018	1.0000000000
p	1.7206000311	1.0000000000
p	0.7544648237	1.0000000000
p	0.3216662007	1.0000000000
d	2.6784400611	1.0000000000
d	0.9225147829	1.0000000000
d	0.3408412315	1.0000000000
f	2.7429299969	1.0000000000
f	0.8957560323	1.0000000000

**Table A19:** Parameters of the cc-TZVP auxiliary basis set of Carbon. Parameters taken from [89].

Shell Type	Exponents	Contraction Coefficients
s	22.2608165950	1.0000000000
s	7.1315246807	1.0000000000
s	3.5380450775	1.0000000000
s	1.2333453175	1.0000000000
s	0.2821517353	1.0000000000
s	0.3468258230	1.0000000000
p	7.2975063903	1.0000000000
p	3.7896065213	1.0000000000
p	1.0633834831	1.0000000000
p	0.2356430320	1.0000000000
p	0.5078423493	1.0000000000
d	9.9000557486	1.0000000000
d	2.3408375066	1.0000000000
d	1.5195338451	1.0000000000
d	0.5788522388	1.0000000000
d	0.3721345858	1.0000000000
f	1.9332589728	1.0000000000
f	1.1560553410	1.0000000000
f	0.4987261239	1.0000000000
g	1.2175667359	1.0000000000

**Table A20:** Parameters of the cc-DZVP auxiliary basis set of Nitrogen. Parameters taken from [89].

Shell Type	Exponents	Contraction Coefficients
s	20.4678978643	1.0000000000
s	7.6243888531	1.0000000000
s	2.3446722210	1.0000000000
s	1.1234062160	1.0000000000
s	0.7258555682	1.0000000000
s	0.3516451521	1.0000000000
p	8.5530798511	1.0000000000
p	2.5349440268	1.0000000000
p	1.0857134625	1.0000000000
p	0.4193736786	1.0000000000
d	3.4384121802	1.0000000000
d	1.2761051199	1.0000000000
d	0.3971760294	1.0000000000
f	3.4010871209	1.0000000000
f	1.3350506486	1.0000000000

**Table A21:** Parameters of the cc-TZVP auxiliary basis set of Nitrogen. Parameters taken from [89].

Shell Type	Exponents	Contraction Coefficients
s	21.6812818892	1.0000000000
s	7.9027065688	1.0000000000
s	2.4447520737	1.0000000000
s	1.2617706294	1.0000000000
s	0.8067419821	1.0000000000
s	0.2885279906	1.0000000000
p	10.3296673020	1.0000000000
p	2.9182107455	1.0000000000
p	1.3834177164	1.0000000000
p	0.7162830530	1.0000000000
p	0.3296257918	1.0000000000
d	13.9094333585	1.0000000000
d	4.5822351942	1.0000000000
d	2.1943496520	1.0000000000
d	0.8349245145	1.0000000000
d	0.4510857395	1.0000000000
f	3.4744937308	1.0000000000
f	1.5532348673	1.0000000000
f	0.8522508678	1.0000000000
g	1.7674440596	1.0000000000

**Table A22:** Parameters of the cc-DZVP auxiliary basis set of Oxygen. Parameters taken from [89].

Shell Type	Exponents	Contraction Coefficients
s	25.5779913844	1.0000000000
s	9.5515670675	1.0000000000
s	2.9409752222	1.0000000000
s	1.3964896911	1.0000000000
s	0.9105756313	1.0000000000
s	0.4821009543	1.0000000000
p	10.8823093197	1.0000000000
p	3.2132775587	1.0000000000
p	1.3802086101	1.0000000000
p	0.4601246170	1.0000000000
d	4.5934895346	1.0000000000
d	1.7871052175	1.0000000000
d	0.4206288858	1.0000000000
f	4.2218855419	1.0000000000
f	1.7894864633	1.0000000000

**Table A23:** Parameters of the cc-TZVP auxiliary basis set of Oxygen. Parameters taken from [89].

Shell Type	Exponents	Contraction Coefficients
s	24.5595006061	1.0000000000
s	8.3254503805	1.0000000000
s	2.8895585562	1.0000000000
s	1.3383587201	1.0000000000
s	0.8797495165	1.0000000000
s	0.2902204697	1.0000000000
p	15.0341204959	1.0000000000
p	3.9838033442	1.0000000000
p	2.2151496463	1.0000000000
p	0.8979637674	1.0000000000
p	0.4128471304	1.0000000000
d	15.8683289847	1.0000000000
d	5.3913486662	1.0000000000
d	2.5385447175	1.0000000000
d	1.0911199995	1.0000000000
d	0.3766843343	1.0000000000
f	4.6812603411	1.0000000000
f	2.1656106741	1.0000000000
f	1.0331835741	1.0000000000
g	2.3079719899	1.0000000000

**Table A24:** Parameters of the cc-DZVP auxiliary basis set of Neon.

Shell Type	Exponents	Contraction Coefficients
s	0.4283083846	1.0000000000
s	0.9127866030	1.0000000000
s	1.4054659820	1.0000000000
s	2.7308004917	1.0000000000
s	10.8821914790	1.0000000000
s	27.4563627600	1.0000000000
p	0.7929884909	1.0000000000
p	2.5008596627	1.0000000000
p	5.9151957867	1.0000000000
p	15.3673109753	1.0000000000
d	0.8283650682	1.0000000000
d	3.3870444721	1.0000000000
d	10.9778901482	1.0000000000
f	3.1170471491	1.0000000000
f	6.8656878672	1.0000000000

**Table A25:** Parameters of the cc-TZVP auxiliary basis set of Neon.

Shell Type	Exponents	Contraction Coefficients
s	0.4218054070	1.0000000000
s	0.9220306716	1.0000000000
s	1.8281445142	1.0000000000
s	4.0122843245	1.0000000000
s	9.3557953735	1.0000000000
s	21.2448698799	1.0000000000
p	0.7819113369	1.0000000000
p	1.2774995450	1.0000000000
p	4.0929391223	1.0000000000
p	6.2303237413	1.0000000000
p	16.9114662817	1.0000000000
d	0.5593906817	1.0000000000
d	1.1997449519	1.0000000000
d	2.6389336208	1.0000000000
d	4.9434479195	1.0000000000
d	14.1003353302	1.0000000000
f	1.5593705359	1.0000000000
f	3.3654768720	1.0000000000
f	8.0058977735	1.0000000000
g	3.9789077704	1.0000000000

**Table A26:** Parameters of the cc-DZVP auxiliary basis set of Argon.

Shell Type	Exponents	Contraction Coefficients
s	0.2021934524	1.0000000000
s	0.5951644570	1.0000000000
s	0.9713367515	1.0000000000
s	2.1348414404	1.0000000000
s	8.3055209987	1.0000000000
s	24.4838599910	1.0000000000
p	0.3806171008	1.0000000000
p	1.0795771281	1.0000000000
p	1.5490430664	1.0000000000
p	4.5409363372	1.0000000000
d	0.4407961817	1.0000000000
d	1.2550515264	1.0000000000
d	5.8159089208	1.0000000000
f	1.2170432674	1.0000000000
f	9.1164484253	1.0000000000

**Table A27:** Parameters of the cc-TZVP auxiliary basis set of Argon.

Shell Type	Exponents	Contraction Coefficients
s	0.2374198920	1.0000000000
s	0.4593140129	1.0000000000
s	0.8759707445	1.0000000000
s	1.6509598569	1.0000000000
s	3.0758784521	1.0000000000
s	5.6934758267	1.0000000000
p	0.2795695538	1.0000000000
p	0.5599111995	1.0000000000
p	1.1757695117	1.0000000000
p	2.6036947304	1.0000000000
p	5.6658077981	1.0000000000
d	0.2898716779	1.0000000000
d	0.6972610882	1.0000000000
d	1.2166500341	1.0000000000
d	2.4487532956	1.0000000000
d	5.4801182100	1.0000000000
f	0.6819926505	1.0000000000
f	1.4331910682	1.0000000000
f	3.3654529504	1.0000000000
g	1.4464903837	1.0000000000
